# Cardiometabolic risk factors and multidomain functional, cognitive, and sensory impairments among tribal adults aged 45 years and above in India: evidence to inform healthy aging policy

**DOI:** 10.3389/fpubh.2026.1795562

**Published:** 2026-03-13

**Authors:** U. Venkatesh, Margubur Rahaman, Varkey Nadakkavukaran Santhosh, Anand Mohan Dixit, Vibha Dutta

**Affiliations:** 1 Department of Community Medicine & Family Medicine, All India Institute of Medical Sciences, Gorakhpur, Uttar Pradesh, India; 2 All India Institute of Medical Sciences, Gorakhpur, Uttar Pradesh, India

**Keywords:** cardiometabolic risk, cognitive impairment, functional impairment, India, public health policy, sensory impairment, tribal health

## Abstract

**Background:**

Evidence on functional, cognitive, and sensory impairments among aging tribal populations in India is inadequate, limiting the effectiveness of ongoing public health policies and programmes aimed at healthy aging and equity. Although national initiatives address non-communicable diseases and primary health care, the integration of functional and cognitive health within these frameworks remains limited. This study aims to assess the burden and determinants of cognitive, functional, and sensory impairments among tribal older-adults and to identify modifiable risk factors that can inform the strengthening of public health policies and healthy aging strategies in tribal populations.

**Materials and methods:**

A community-based cross-sectional study prospectively collected data using complete enumeration across five tribal villages of Gorakhpur district, north India. Older-adults aged 45 years and above were considered as study participants to assessed the mobility limitation, activities of daily living (ADL) limitation, memory, visual, and hearing impairment. Cardiometabolic and anthropometric indicators were the primary explanatory variables. Descriptive statistics, bivariate analyses, and multivariable logistic regression models were applied.

**Results:**

Among 788 tribal older-adults, the prevalence of mobility limitation was 18.2%, ADL limitation 28.8%, memory impairment 17.5%, visual impairment 20.6%, and hearing impairment 20.8%. Hypertension significantly increased the adjusted odds ratio (aOR) of ADL limitation (aOR 2.08) and memory impairment (aOR 2.31). Overweight status predicted mobility limitation (aOR 1.57), memory impairment (aOR 1.88), visual impairment (aOR 1.65), and hearing impairment (aOR 1.67). Adults aged 60–69 years had substantially higher odds of mobility limitation (aOR 6.02) and memory impairment (aOR 7.48) compared with those aged 45–59 years.

**Conclusion:**

Functional, cognitive, and sensory impairments were common among tribal older-adults and were strongly associated with hypertension, overweight, and advancing age. These findings highlight modifiable cardiometabolic pathways contributing to early functional and cognitive decline. Strengthening healthy aging strategies within existing primary care programmes by optimizing routine multidomain screening in tribal settings may improve early detection and reduce disability burden.

## Background

Advancing age in resource-constrained settings poses an increasing public health concern, especially for socioeconomically disadvantaged populations whose cumulative life-course adversities extend into older adulthood (1). Functional, cognitive, and sensory limitations, though commonly linked to biological aging, are progressively evident beginning in midlife (2). The early emergence of these impairments is especially concerning, as it significantly increases the risks of disability, diminished independence, poorer quality of life, and midlife mortality (3). Concurrently, the increasing prevalence of non-communicable diseases (NCDs), such as hypertension, diabetes, and obesity, is identified as a key contributor to functional and cognitive deterioration (4). Cardiometabolic risk factors hasten age-related decline via mechanisms involving vascular injury, inflammation, neuropathy, and reduced physiological reserve (5).

Recent evidence from India highlights pronounced social-group disparities in functional, cognitive, and sensory impairments. Tribal communities, comprising 8.6% of the population, demonstrate a markedly higher risk of cognitive impairment than non-tribal groups (6). Vision impairment also appears more prevalent among tribal older adults than non-tribal counterparts (43% vs. 33%) (7), although differences in hearing impairment remain inconsistent (8). Current evidence adequately compares tribal and non-tribal older populations regarding functional, cognitive, and sensory impairments; however, it has largely neglected variations within tribal aging groups across sociodemographic, economic, and health factors.

Despite this, public health research on healthy aging within tribal population in India remains limited (9, 10). Addressing these gaps is critical for identifying potentially modifiable pathways and for informing the design and implementation of equitable public health policies. By generating population-based evidence from an underserved tribal setting, the present study contributes directly to public health policy by highlighting priority risk factors that can be addressed through existing national programmes, including the National Programme for Prevention and Control of Cancer, Diabetes, Cardiovascular Diseases and Stroke (NPCDCS), Ayushman Bharat–Health and Wellness Centres (AB_HWCs), and the Tribal Health Action Plan under the National Health Mission.

Against this backdrop, the present study examines the prevalence and determinants of cognitive, functional, and sensory impairments among tribal adults aged 45 years and above in Gorakhpur district, Uttar Pradesh, with particular emphasis on cardiometabolic and anthropometric risk profiles. By extending the analytical focus to adults from midlife onward, the study adopts a preventive public health perspective, recognizing that early identification of functional and cognitive vulnerability is essential for strengthening healthy aging trajectories. Generating population-based evidence from an underserved tribal setting, the findings aim to inform public health policy by identifying modifiable risk pathways and highlighting opportunities to integrate multidomain geriatric screening within existing primary care and tribal health frameworks. These insights contribute to strengthening healthy aging strategies and advancing equitable geriatric service delivery among tribal populations.

## Materials and methods

### Study setting

**Figure 1:**
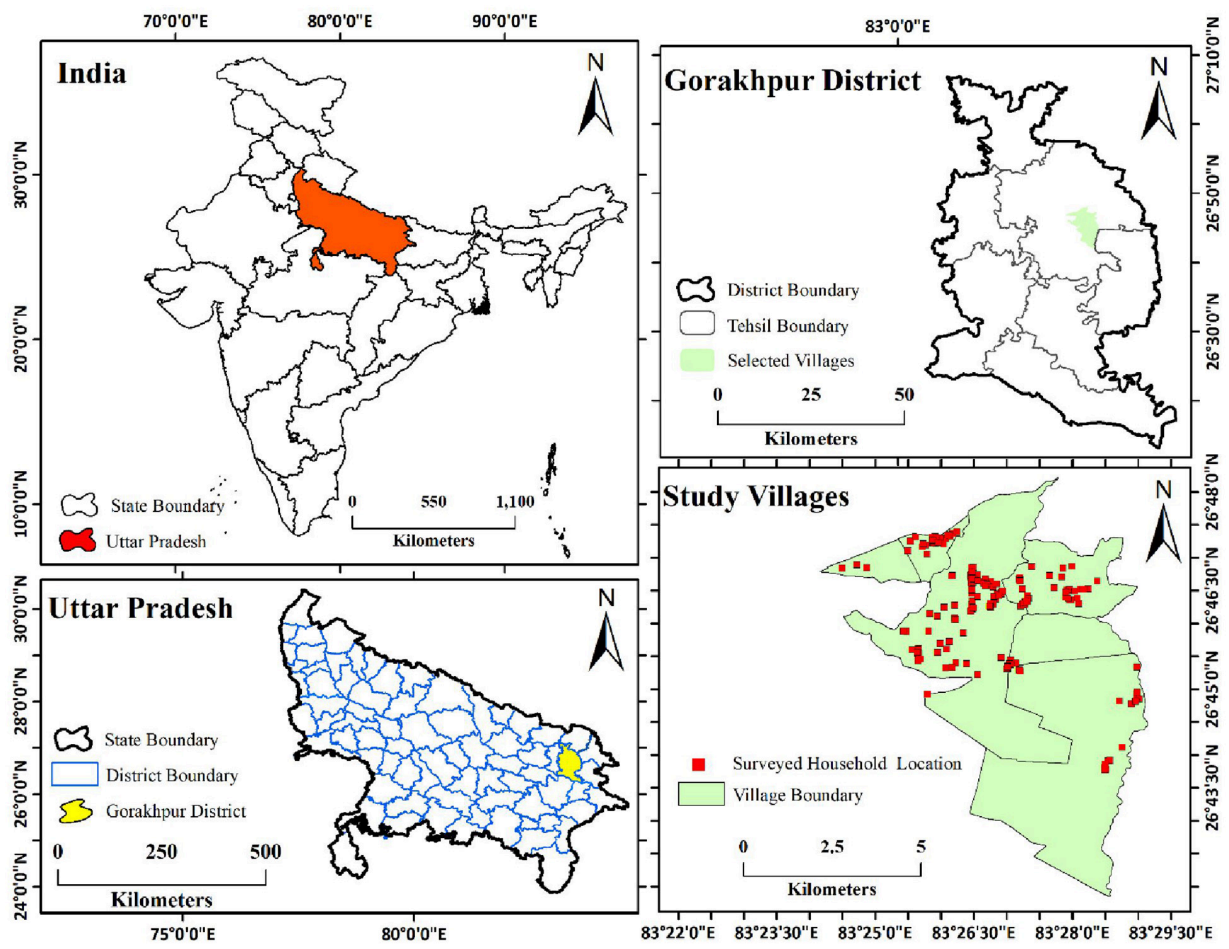
Location of the study area, Gorakhpur, India.

The study was carried out in five tribal villages in the Gorakhpur sub-division of Uttar Pradesh, India. The region lies between 83°23′30″-83°29′15″E longitude and 26°40′15″-26°48′15″N latitude (Figure 1). The region is characterized by flat alluvial terrain and a monsoon climate. According to the Census of India (2011), the wider Gorakhpur district has a population of 4.44 million, of whom tribal communities constitute only 0.4% (11), resulting in limited representation in large-scale surveys. Consequently, empirical evidence on tribal aging health in this region remains limited. To bridge this gap, the study examined tribal-inhabited villages in the Gorakhpur sub-division, allowing a focused assessment of health characteristics among a demographically marginal and understudied population.

### Study population

The “*Vantangiya*” tribal community, a historically migrant group selected as study population. In 1922, the British colonial administration resettled them in the study area to assist afforestation on government land. The term “*Vantangiya*,” derived from van (forest), denotes their enduring connection to forest-based labor and livelihoods.

### Study design

A cross-sectional study design. This community-based cross-sectional study prospectively collected primary data during household visits to estimate the prevalence and examine the cardiometabolic correlates of functional, cognitive, and sensory impairments among tribal older adults. Exposures and outcomes were measured concurrently, permitting assessment of distribution and associations but not causal inference. The design was appropriate for geographically remote tribal settings where longitudinal follow-up and reliable records are limited. Complete household enumeration minimized selection bias, while standardized instruments, trained interviewers, and objective cardiometabolic measurements reduced potential recall and social desirability bias.

### Data collection

A complete household enumeration was conducted in the five selected villages from March to June 2024. All residents aged 18 years and above were eligible to participate. Overall, 4,000 tribal individuals were enumerated and interviewed on sociodemographic, economic, clinical, and disease-related variables. For this analysis, only those aged 45 years and above were included, totaling a final sample of 788 participants.

### Survey tools and procedures

Data were gathered using a structured questionnaire covering three domains: household sociodemographic characteristics, clinical profile, and health status. Functional, cognitive, and sensory impairments were evaluated through self-reported items consistent with established measures in aging and public health research (8, 11, 12). Cardiometabolic and anthropometric parameters were recorded using standardized protocols, including blood pressure measurement, random blood glucose testing, and assessment of height, weight, waist, and hip circumference (11).

### Outcome variables

Mobility limitation, Activities of Daily Living (ADL) limitation, memory impairment, and visual and hearing impairment were considered as outcome variables. All outcomes were derived from self-reported responses and were coded dichotomously (no = 0; yes = 1). Mobility limitation was assessed using a self-reported item asking whether the respondent usually experienced imbalance while walking or standing (13). ADL limitation was measured using a standard set of questions assessing difficulty in performing basic daily activities, including bathing, dressing, eating, getting in or out of bed, using the toilet, and walking across a room (14). Responses to each item were recorded as yes (1) or no (0). A composite ADL limitation variable was created, with respondents reporting difficulty in at least one activity classified as having an ADL limitation (none = 0; any = 1) (12). Memory impairment was assessed using a self-reported screening item asking whether the respondent usually experienced forgetting. Participants responding affirmatively were classified as having memory impairment (11). Visual and hearing impairment was assessed by asking respondents whether they had ever been diagnosed with any eye or vision problem or condition (including near- or farsightedness) and diagnosed with any hearing- or ear-related problem, respectively. Measures of mobility limitation, ADL limitation, visual impairment, and hearing impairment were adapted from the Longitudinal Ageing Study in India (LASI) instruments, which are validated for assessing functional status among the older population in India (15). Memory impairment was measured using a self-reported item previously validated and employed in population-based studies (11).

### Explanatory variables

Based on existing aging studies (2, 8, 11, 12), a range of factors were selected as explanatory variables. Cardiometabolic and anthropometric indicators were considered as key predictors. In the cardiometabolic indicators, blood pressure was measured three times, and the average of the three measurements was considered the final blood pressure value for analysis. Blood pressure readings were categorized as normal (< 120/ < 80 mmHg), pre-hypertension (120–139/80–89 mmHg), and hypertension (140–159/90–99 or ≥160/≥100 mmHg). Random blood sugar (RBS) was measured using a digital glucometer with a finger-prick blood sample obtained via test strips. The RBS was categorized as non-diabetic (< 140 mg/dL), prediabetic (140–199 mg/dL), and diabetic (>199 mg/dL). Anthropometric indicators included body-mass index (BMI) and waist-hip ratio (WHR). BMI was calculated using measured height and weight following standard operating procedures (SoP). The BMI was grouped into four categorized (normal, underweight, overweight, and obese) following the WHO Asian cut-offs. WHR was measured using waist and hip circumferences obtained with a measuring tape following SoP. WHR was calculated in accordance with WHO Asian guidelines and classified as normal or high (abdominal obesity) using sex-specific thresholds (≥1.0 for men and ≥0.90 for women).

Respondent's age (45–59, 60–69, 70–79, and ≥80 years), gender (male, female), and level of education (illiterate, primary education, and secondary or above), occupation (unemployed, unpaid labor, waged labor, and other) and diet (non-vegetarian or vegetarian) and any substance use (no=0, and yes=1) considered as covariates. Substance use was constructed using responses to multiple questions assessing current use of psychoactive substances. These included current smoking of tobacco, current use of smokeless tobacco (such as *gutkha, pan masala, and khaini*), current alcohol consumption, and current use of any other substances (16). Each item was coded as yes (1) or no (0), and a cumulative score was generated by summing responses across the four items. Participants with a score of 0 were classified as non-users, while those with a score of 1–4 were classified as any substance users (16).

### Statistical analysis

A series of statistical analyses were applied to examine the prevalence and predictors of functional, cognitive, and sensory impairments. Participant characteristics were summarized using descriptive statistics proportions with 95% confidence intervals (CIs). Prevalence was calculated for each health outcome. Bivariate analyses were performed to present prevalence of health outcomes by respondent's backgrounds. Pearson chi-square and Fisher's exact test were applied to assess the significant level of bivariate association. In bivariate analyses, the Fisher's exact test applied when expected cell counts were less than five. A significance level of *p* < 0.05 was considered statistically significant. Finally, five separate multivariable binary logistic regression models were performed to estimate adjusted likelihood of each outcomes: mobility limitation (no = 0, and yes = 1), any ADL limitation (no = 0, and yes = 1), memory impairment (no = 0, and yes = 1), visual impairment (no = 0, and yes = 1), and hearing loss (no = 0, and yes = 1). In these five regression models, blood pressure, RBS, BMI, and WHR were selected as key explanatory variables. Where, age group, gender, education level, occupation, diet type, substance used as covariates to estimate adjusted association between outcome and key explanatory variables applying following equation:


$$\begin{array}{l} log=\left(\frac{p_{i}}{1-p_{i}}\right)=\beta_{0}+\beta_{1}BP_{i}+\beta_{2}RBG_{i}+\beta_{3}BMI_{i}+\beta_{4}WHR_{i}\\ \;+\beta_{5}Age_{i}+.........+\beta_{10}Subtance\;use_{i} \end{array}$$

Where *p*_*i*_ denotes the probability that individual *i* has the specified impairment or limitation (mobility limitation, any ADL limitation, memory impairment, visual impairment, and hearing impairment). β_1_...β_10_ indicates explanatory variables.

Multivariable logistic regression model's results were presented in adjusted odds ratios (aORs) with 95% CIs. Multicollinearity was evaluated using variance inflation factor (VIF) tests, which ensured no substantial multicollinearity among the explanatory variables. Multivariable model fit was evaluated using the likelihood ratio chi-square (χ^2^) statistic and pseudo-R^2^. To assess discriminative ability, we generated receiver operating characteristic (ROC) curves and reported the area under the curve (AUC) for each model. All analyses were performed using Stata software 17 (StataCorp, College Station, TX).

## Result

### Participant characteristics

Out of total 788 tribal older adults, nearly half of them were aged 45–59 years (45.3%), followed by 60–69 years (44.8%), and ≥70 years (9.9%). About 47% and 53% of them were women and men, respectively (Table 1). Low levels of educational was predominant among them, with 86.2% reported no formal schooling and only 3.8% completed secondary or higher education. One-third of older-adult were engaged in household unpaid work (31.7%), while 22.7% reported being unemployed. Non-vegetarian diet (72.2%) observed to be common among study population. About 17.6% of the older-adults reported use of any substances.

**Table 1:** Sociodemographic profile and prevalence of functional, cognitive, and sensory impairments among tribal adults aged 45 years and above in Gorakhpur, India.

**Characteristic**	**Category**	***n***	**Percent**	**95% CI**
	Mobility limitation	143	18.1	15.6–21.0
	ADL limitation	227	28.8	25.7–32.1
Health status	Memory impairment	138	17.5	15.0–20.3
	Visual impairment	162	20.6	17.9–23.5
	Hearing impairment	164	20.8	18.1–23.8
Age group	45–59 years	357	45.3	41.9–48.8
	60–69 years	353	44.8	41.4–48.3
	70–79 years	62	7.9	6.2–10.0
	≥80 years	16	2	1.2–3.3
Gender	Male	417	52.9	49.4–56.4
	Female	371	47.1	43.6–50.6
Education level	Illiterate	679	86.2	83.6–88.4
	Up to primary	79	10	8.1–12.3
	Secondary or above	30	3.8	2.7–5.4
Occupation	Unemployed	179	22.7	19.9–25.8
	Unpaid labor	250	31.7	28.6–35.1
	Waged labor	197	25	22.1–28.1
	Other	162	20.6	17.9–23.5
Diet	Non-vegetarian	569	72.2	69.0–75.2
	Vegetarian	219	27.8	24.8–31.0
RBS category	Non-diabetic	323	65.1	60.8–69.2
	Prediabetic	148	29.8	26.0–34.0
	Diabetic	25	5.0	3.4–7.4
	Missing^*^	292	–	–
BMI category	Normal	399	50.6	47.1–54.1
	Underweight	70	8.9	7.1–11.1
	Overweight	213	27	24.0–30.3
	Obese	106	13.5	11.2–16.0
WHR category	Normal	220	27.9	24.9–31.2
	High	568	72.1	68.8–75.1
Any substance use	No	649	82.4	79.5–84.8
	Yes	139	17.6	15.1–20.4

CI, Confidence Interval; ^*^individuals without RBS records.

### Multidomain functional and cognitive health outcomes

**Figure 2:**
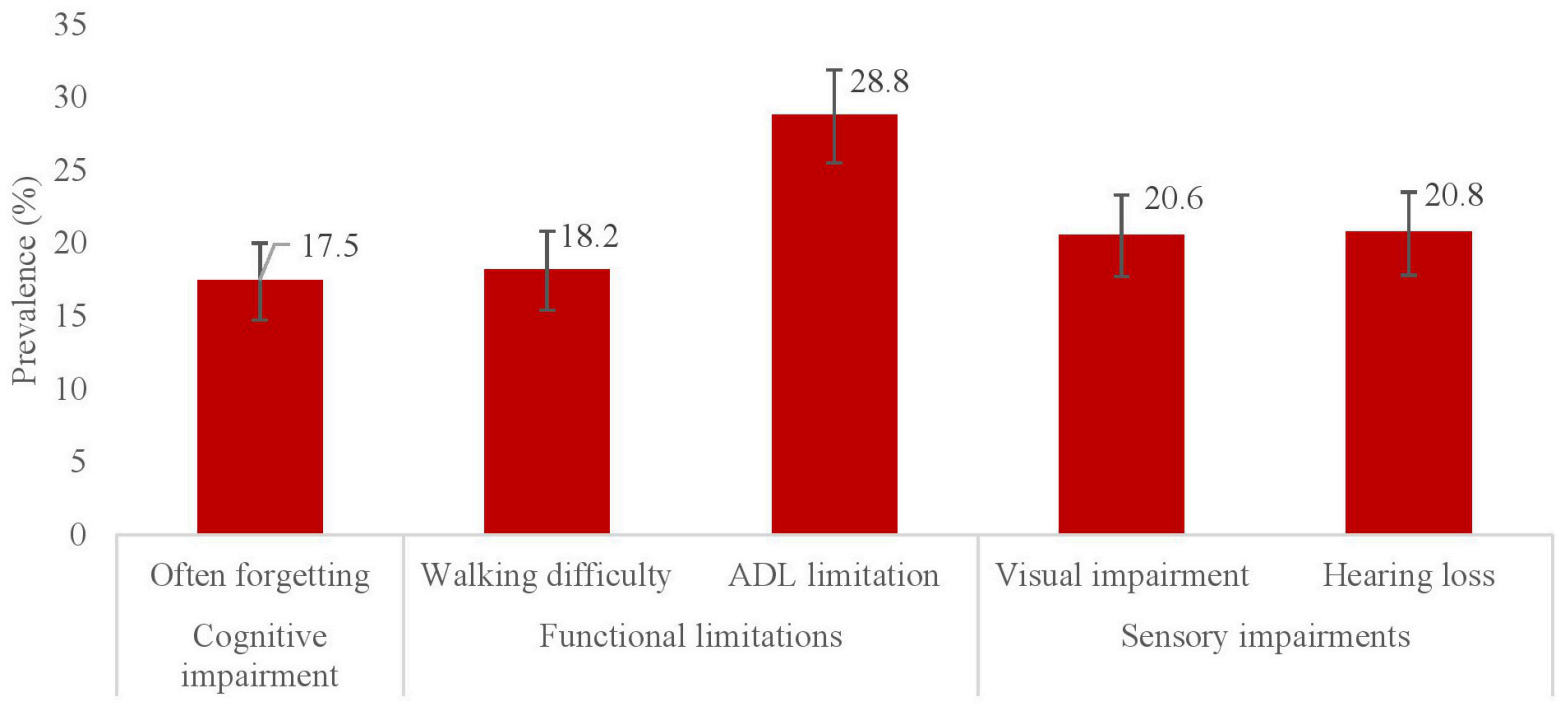
Prevalence of functional, cognitive, and sensory impairments among tribal older adults (≥45 years), Gorakhpur.

Of 788 older adult tribal participants, 17.5% (95% CI: 15.0–20.3) of them reported memory impairment (Figure 2). The prevalence of mobility and any ADL limitation were 18.2% (95% CI: 15.6–21.0) and 28.8% (95% CI 25.7–32.1), respectively. Sensory impairments were also prevalent among the study participants. In particular, visual impairment was reported by 20.6% (95% CI: 17.9–23.5) and hearing impairment by 20.8% (95% CI: 18.1–23.8) of the older adults.

### Distribution of cardiometabolic and anthropometric risk factors

**Figure 3:**
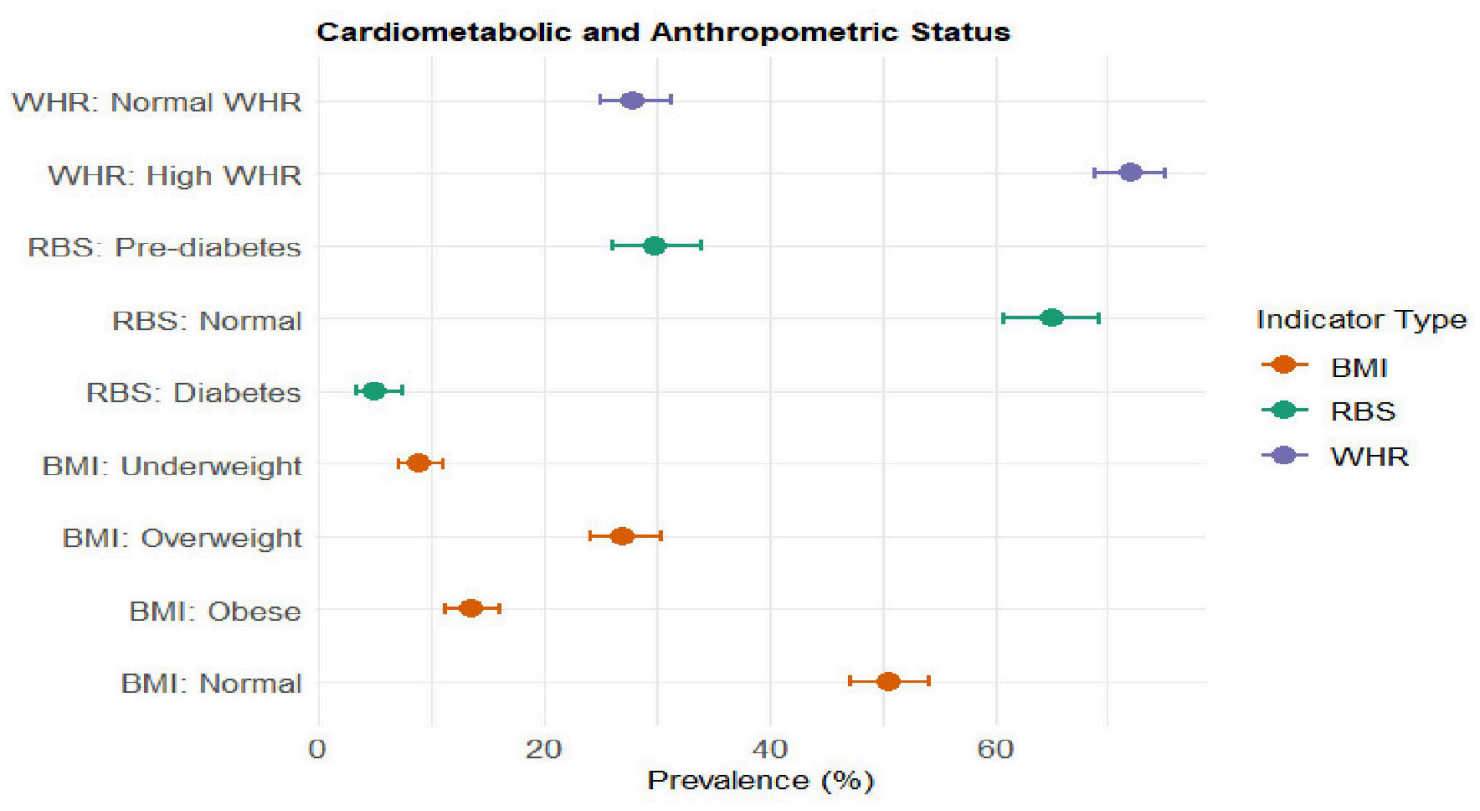
Prevalence of cardiometabolic and anthropometric risk among tribal older adults (≥45 years), Gorakhpur.

More than half of older adults were non-diabetic (65.1%; 95% CI: 60.8–69.2), while 29.8% (95% CI: 26.0–34.0) were pre-diabetic and 5.0% (95% CI: 3.4–7.4) diabetic (Figure 3). Regarding BMI, 50% (95% CI: 47.1–54.1) of them were belonged to normal category. The prevalence of overweight, obese, and underweight was 27% (95% CI: 24.0–30.3), 13.5% (95% CI: 11.2–16.0), and 8.9% (95% CI: 7.1–11.1), respectively. Central adiposity was highly prevalent among the study participants. About 72.1% of study participants had high WHR (95% CI: 68.8–75.1), where only 27.9% (95% CI: 24.9–31.2) had normal WHR (Figure 3).

### Bivariate distribution of multidomain impairments by cardiometabolic and anthropometric risk status

Bivariate analyses found a significant variation in functional, cognitive, and sensory impairments across cardiometabolic and anthropometric backgrounds (Table 2). Compared with older adults with normal blood pressure, those with hypertension demonstrated higher prevalence of any ADL limitation (38.3% vs. 20.0%), memory impairment (22.0% vs. 9.4%), visual impairment (26.0% vs. 12.9%), and hearing impairment (26.3% vs. 18.8%). Similarly, pre-diabetic older adults reported higher levels of any ADL limitation (37.8% vs. 24.5%), memory impairment (26.4% vs. 17.0%), visual impairment (30.4% vs. 16.7%), and hearing impairment (26.4% vs. 15.8%) compared with non-diabetic counterparts (*p* ≤ 0.012). BMI differences were most notable for memory impairment, which was more prevalent among underweight (24.3%) and overweight older-adults (22.5%) than those with normal BMI (15%; *p* = 0.022) counterparts. Visual impairment was significantly varied with WHR, which was higher among persons with high WHR (22.4% vs. 15.9%; *p* = 0.044).

**Table 2:** Distribution of multidomain impairments by cardiometabolic and anthropometric risk status among tribal adults aged 45 years and above, Gorakhpur, India.

**Cardiometabolic indicator**	**Total (*n*)**	**Mobility limitation *n* (%)**	***p* value**	**ADL limitation *n* (%)**	***p* value**	**Memory impairment *n* (%)**	***p* value**	**Visual impairment *n* (%)**	***p* value**	**Hearing impairment *n* (%)**	***p* value**
**Blood pressure**			0.051		**0.001**		**0.012**		**0.007**		**0.011**
Normal	85	10 (11.8)		17 (20.0)		8 (9.4)		11 (12.9)		16 (18.8)	
Pre-hypertension	403	67 (16.6)		95 (23.6)		64 (15.9)		73 (18.1)		69 (17.1)	
Hypertension	300	66 (22.0)		115 (38.3)		66 (22.0)		78 (26.0)		79 (26.3)	
**RBS**			0.053^*^		**0.001** ^*^		**0.012** ^*^		**0.008** ^*^		**0.012** ^*^
Normal	323	54 (16.7)		79 (24.5)		55 (17.0)		54 (16.7)		51 (15.8)	
Pre-diabetes	148	37 (25.0)		56 (37.8)		39 (26.4)		45 (30.4)		39 (26.4)	
Diabetes	25	4 (16.0)		6 (24.0)		4 (16.0)		8 (32.0)		7 (28.0)	
**BMI**			0.075		0.508		**0.022**		0.168		0.197
Normal	399	69 (17.3)		108 (27.1)		60 (15.0)		73 (18.3)		77 (19.3)	
Underweight	70	13 (18.6)		25 (35.7)		17 (24.3)		19 (27.1)		17 (24.3)	
Overweight	213	49 (23.0)		62 (29.1)		48 (22.5)		51 (23.9)		53 (24.9)	
Obese	106	12 (11.3)		32 (30.2)		13 (12.3)		19 (17.9)		17 (16.0)	
**WHR**			0.222		0.554		0.173		**0.044**		0.184
Normal	220	34 (15.5)		60 (27.3)		32 (14.6)		35 (15.9)		39 (17.7)	
High	568	109 (19.2)		167 (29.4)		106 (18.7)		127 (22.4)		125 (22.0)	

^*^Fisher's exact test; other significant tests were performed based on chi-square test. Values in bold indicate statistically significant.

### Bivariate distribution of multidomain impairments by sociodemographic characteristics

Functional, memory, and sensory impairments found to be substantially varied across demographic and socioeconomic backgrounds (Table 3). Compared with those aged 45–59 years, adults aged 60–69 years and 70–79 years showed substantially higher prevalence of mobility limitation (29.2% and 25.8% vs. 5.9%) and any ADL limitations (42.8% and 37.1% vs. 13.2%). Memory impairment increased steeply with age (56.3% among ≥80 age group vs. 5.9% in 45–59 age group). A similar age gradient in sensory impairments also observed, where visual impairment rose from 4.5% in 45–59 age group to 41.9% among aged 70–79 years, while hearing impairment increased from 5% to 43.8% across the same age range (*p* ≤ 0.001). Women were more likely than men to have visual impairment (24.3% vs. 17.3%, *p* = 0.015). Mobility limitation was more prevalent among illiterates (19.3%) compared with those having secondary education (3.3%, *p* = 0.040). Unemployed persons demonstrated the highest prevalence of mobility limitation (25.7%) and ADL limitations (34.1%) compared with unpaid and wage laborers. Unemployed persons also were shown higher prevalence of visual impairment (26.8%) and hearing impairment (29.6%) (*p* < 0.05) than their counterparts. Non-vegetarian older adults had a significantly higher prevalence of mobility limitation compared with vegetarians (20.2% vs. 12.8%, *p* = 0.015), whereas vegetarians showed a higher prevalence of visual impairment (25.6% vs. 18.6%, *p* = 0.031).

**Table 3:** Multidomain impairment prevalence across sociodemographic groups among tribal adults aged 45 years and above, Gorakhpur, India.

**Characteristic**	**Total (n)**	**Mobility limitation *n* (%)**	***p* value**	**ADL limitation *n* (%)**	***p* value**	**Memory impairment *n* (%)**	***p* value**	**Visual impairment *n* (%)**	***p* value**	**Hearing impairment *n* (%)**	***p* value**
**Age group**			**≤0.001** ^*^		**≤0.001** ^*^		**≤0.001** ^*^		**≤0.001** ^*^		**≤0.001** ^*^
45–59 years	357	21 (5.9)		47 (13.2)		21 (5.9)		16 (4.5)		18 (5.0)	
60–69 years	353	103 (29.2)		151 (42.8)		94 (26.6)		113 (32.0)		113 (32.0)	
70–79 years	62	16 (25.8)		23 (37.1)		14 (22.6)		26 (41.9)		26 (41.9)	
≥80 years	16	3 (18.8)		6 (37.5)		9 (56.3)		7 (43.8)		7 (43.8)	
**Gender**			0.423		0.768		0.345		**0.015**		0.624
Male	417	80 (19.2)		122 (29.3)		68 (16.3)		72 (17.3)		84 (20.1)	
Female	371	63 (17.0)		105 (28.3)		70 (18.9)		90 (24.3)		80 (21.6)	
**Education level**			**0.040** ^*^		0.324^*^		0.415^*^		0.166^*^		0.145^*^
Illiterate	679	131 (19.3)		198 (29.2)		124 (18.3)		147 (21.7)		149 (21.9)	
Up to primary	79	11 (13.9)		24 (30.4)		11 (13.9)		12 (15.2)		12 (15.2)	
Secondary or above	30	1 (3.3)		5 (16.7)		3 (10.0)		3 (10.0)		3 (10.0)	
**Occupation**			**≤0.001**		**0.029**		0.841		**0.005**		**0.007**
Unemployed	179	46 (25.7)		61 (34.1)		35 (19.6)		48 (26.8)		53 (29.6)	
Unpaid labor	250	34 (13.6)		67 (26.8)		44 (17.6)		58 (23.2)		47 (18.8)	
Waged labor	197	25 (12.7)		44 (22.3)		33 (16.8)		25 (12.7)		31 (15.7)	
Other	162	38 (23.5)		55 (34.0)		26 (16.1)		31 (19.1)		33 (20.4)	
**Diet type**			**0.015**		0.055		0.184		**0.031**		0.781
Non-vegetarian	569	115 (20.2)		153 (26.9)		106 (18.6)		106 (18.6)		117 (20.6)	
Vegetarian	219	28 (12.8)		74 (33.8)		32 (14.6)		56 (25.6)		47 (21.5)	
**Any substance use**											
No	649	128 (19.7)	0.013	192 (29.6)		110 (16.9)		139 (21.4)	0.197	143 (22.0)	0.068
Yes	139	15 (10.8)		35 (25.2)		28 (20.1)		23 (16.5)		21 (15.1)	

^*^Fisher's exact test; other significant tests were performed based on chi-square test. Values in bold indicate statistically significant.

### Multivariable logistic regression analysis of multidomain impairments

**Figure 4:**
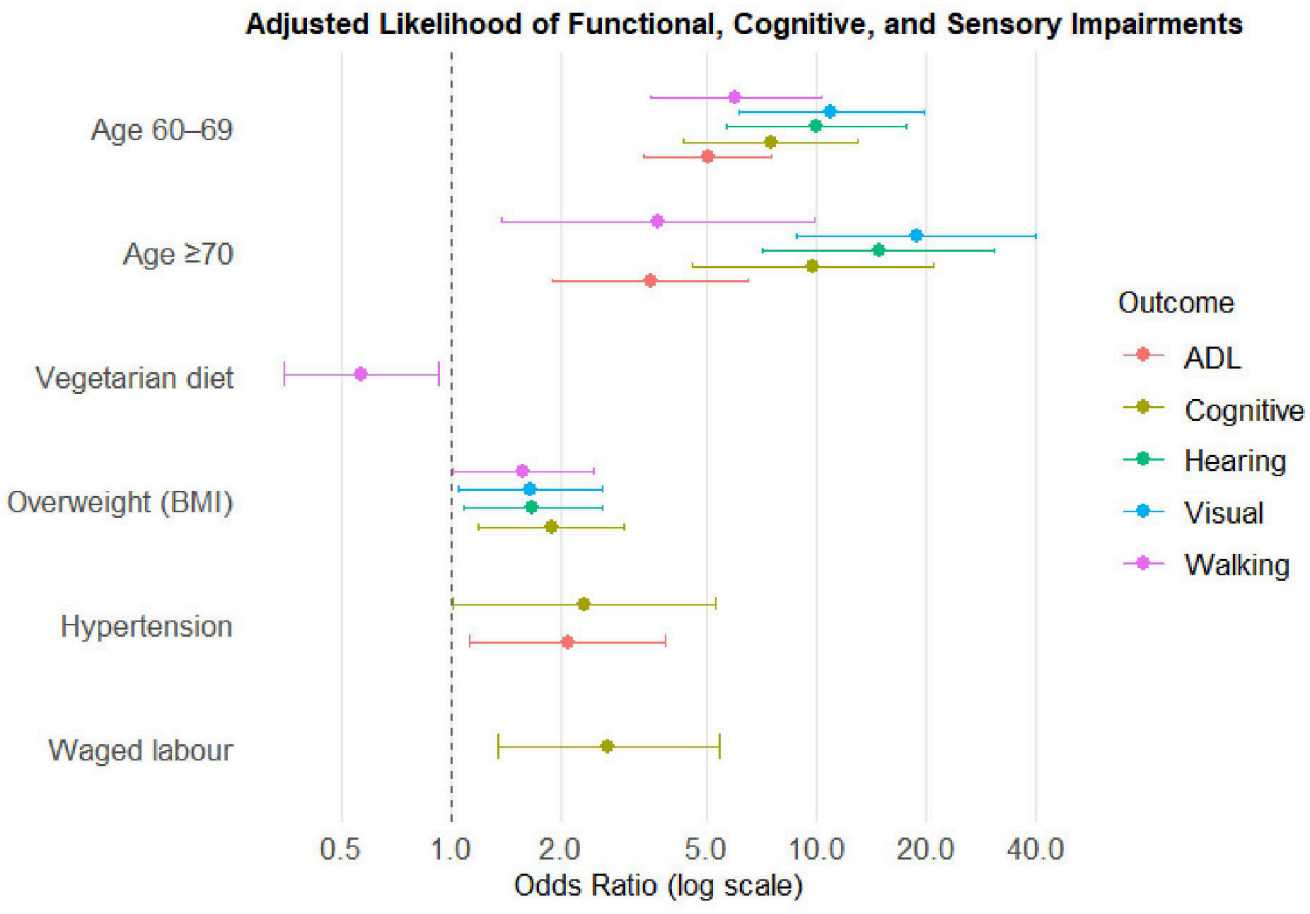
Adjusted odds ratio for functional, cognitive, and sensory impairments among tribal older adults (≥45 years), Gorakhpur.

Overall, the multivariable logistic regression results indicate that hypertension, overweight were the consistent and significant predictors of functional limitation, memory, and sensory impairments (Figure 4). Cardiometabolic and anthropometric indicators showed significant associations with functional, cognitive, and sensory impairments. Hypertension was significantly associated with higher likelihood of any ADL limitation (aOR 2.08; 95% CI 1.12–3.86) and memory impairment (aOR 2.31; 95% CI 1.01–5.27), compared with normal blood pressure. Overweight status found to be significantly associated with mobility limitation (aOR 1.57; 95% CI 1.01–2.44), memory impairment (aOR 1.88; 95% CI 1.19–2.97), visual (aOR 1.65; 95% CI 1.05–2.60), and hearing impairment (aOR 1.67; 95% CI 1.08–2.59).

Age, occupation, diet and substance use also found as significant predictors of functional, cognitive, and sensory impairments. Older adults aged 60–69 years had substantially higher odds of mobility limitation (aOR: 6.02; 95% CI: 3.52–10.28), ADL limitation (aOR: 5.04; 95% CI: 3.36–7.56), memory impairment (aOR: 7.48; 95% CI: 4.31–12.99), visual impairment (aOR: 10.99; 95% CI: 6.14–19.65), and hearing impairment (aOR: 9.96; 95% CI: 5.66–17.52) compared with the 45–59 age group (Table 4). The likelihood increased even further for those aged ≥70 years, with odds ratios ranging from 3.51 for ADL limitation (95% CI: 1.89–6.52) to 18.73 for visual impairment (95% CI: 8.82–39.78). Occupational status shown marginal association. For instance, individuals engaged in waged labor demonstrated higher odds of memory impairment (aOR: 2.68; 95% CI: 1.34–5.39). Dietary pattern also showed a significant association with functional limitations. Vegetarian participants had lower odds of mobility limitation (aOR: 0.57; 95% CI: 0.35–0.92) compared with non-vegetarians (Table 4). The multivariable logistic regression model predicting mobility limitation (AUC: 0.74), ADL limitations (AUC: 0.72), memory impairment (AUC: 0.76), visual impairment (AUC: 0.79), and hearing loss (AUC: 0.77) demonstrated good discriminative ability (Table 4).

**Table 4:** Multivariable associations of cardiometabolic and anthropometric risk factors with multidomain impairments among tribal adults aged ≥45 years in Gorakhpur, India.

**Predictor**	**Category**	**Model I: Mobility Limitation aOR (95% CI)**	**Model II: Any ADL Limitation aOR (95% CI)**	**Model III: Memory Impairment aOR (95% CI)**	**Model IV: Visual Impairment aOR (95% CI)**	**Model V: Hearing Impairment aOR (95% CI)**
Blood Pressure (Ref: Normal)	Pre-hypertension	1.53 (0.72–3.24)	1.19 (0.65–2.18)	1.79 (0.79–4.04)	1.50 (0.72–3.13)	0.85 (0.44–1.63)
	Hypertension	1.89 (0.88–4.06)	2.08 (1.12–3.86)^*^	2.31 (1.01–5.27)^*^	1.74 (0.82–3.68)	1.16 (0.59–2.26)
RBS (Ref: Normal)	Prediabetic/diabetic	1.25 (0.76–2.07)	1.27 (0.82–1.98)	1.23 (0.75–2.04)	1.58 (0.96–2.59)	1.38 (0.83–2.27)
	Missing	0.90 (0.56–1.43)	1.08 (0.73–1.60)	0.69 (0.42–1.12)	0.89 (0.56–1.42)	1.42 (0.91–2.23)
BMI (Ref: Normal)	Underweight	1.07 (0.53–2.14)	1.25 (0.70–2.23)	1.80 (0.92–3.51)	1.39 (0.72–2.66)	1.08 (0.56–2.09)
	Overweight	1.57 (1.01–2.44)^*^	1.16 (0.78–1.72)	1.88 (1.19–2.97)^*^	1.65 (1.05–2.60)^*^	1.67 (1.08–2.59)^*^
	Obese	0.70 (0.35–1.41)	1.27 (0.76–2.13)	0.90 (0.45–1.79)	1.07 (0.58–1.99)	0.90 (0.48–1.69)
WHR (Ref: Normal)	High WHR	0.99 (0.61–1.62)	0.83 (0.55–1.26)	0.93 (0.56–1.54)	0.94 (0.58–1.54)	0.87 (0.54–1.40)
Age (Ref: 45–59)	60–69	6.02 (3.52–10.28)^**^	5.04 (3.36–7.56)^**^	7.48 (4.31–12.99)^**^	10.99 (6.14–19.65)^**^	9.96 (5.66–17.52)^**^
	≥70	4.25 (2.00–9.02) ^**^	3.51 (1.89–6.52)^**^	9.81 (4.59–20.97)^**^	18.73 (8.82–39.78)^**^	14.82 (7.13–30.78)^**^
Gender (Ref: Male)	Female	1.03 (0.57–1.86)	1.05 (0.62–1.77)	1.69 (0.89–3.19)	1.48 (0.81–2.70)	1.25 (0.70–2.23)
Education (Ref: Illiterate)	Formal educated	0.68 (0.35–1.34)	1.16 (0.69–1.92)	0.97 (0.50–1.85)	0.92 (0.48–1.77)	0.83 (0.44–1.56)
Occupation (Ref: Unemployed)	Unpaid labor	0.67 (0.37–1.21)	1.06 (0.63–1.77)	1.29 (0.71–2.35)	1.51 (0.86–2.65)	0.97 (0.55–1.71)
	Waged labor	0.90 (0.46–1.76)	1.18 (0.66–2.11)	2.68 (1.34–5.39)	1.38 (0.70–2.72)	1.53 (0.80–2.93)
	Other	1.03 (0.58–1.82)	1.22 (0.72–2.04)	1.13 (0.59–2.15)	1.01 (0.56–1.84)	0.84 (0.47–1.49)
Diet (Ref: Non-Veg)	Vegetarian	0.57 (0.35–0.92)^*^	1.36 (0.94–1.96)	0.65 (0.41–1.05)	1.44 (0.95–2.21)	0.97 (0.63–1.49)
Any substance use (Ref: No)	Yes	0.40 (0.32–1.75)	0.73 (0.45–1.18)	1.24 (0.70–2.21)	1.10 (0.66–1.78)	1.23 (73–2.14)
Constant	—	0.05 (0.02–0.14)^*^	0.08 (0.03–0.18)^*^	0.015 (0.005–0.045)^*^	0.014 (0.005–0.039)^*^	0.034 (0.013–0.089)^*^
AUC	—	0.74	0.72	0.76	0.79	0.77

*Ref*, Reference; CI, Confidence Interval; aOR, Adjusted Odds Ratio; ^**^p < 0.001; ^*^p < 0.01.

## Discussion

This study contributes to geriatric health research by providing population-based evidence on functional, cognitive, and sensory impairments among tribal adults from midlife onward- a group under-represented in national aging studies. By showing that cardiometabolic and anthropometric risks, particularly hypertension and overweight, are consistently associated with impairments across multiple domains, the findings highlight early functional and cognitive vulnerability in tribal populations. The results align with national programmes such as NPCDCS, AB-HWCs, and the Tribal Health Action Plan, emphasizing the need to integrate routine functional, cognitive, and sensory screening into HWCs. Overall, the study offers actionable insights to strengthen geriatric prevention and care within existing public health systems serving tribal communities.

Although present literature exhibited a considerable burden of mobility limitations, ADL disability, memory, and visual impairment, these prevalences were lower than those reflected in national prevalence estimates for tribal older adults (6, 8, 12). In contrast, the prevalence of hearing impairment was significantly higher than national estimates among tribal aging populations (8). These findings extend the current evidence base by demonstrating that regional morbidity patterns among tribal older adults may differ substantially from national estimates, thereby underscoring the limitations of relying exclusively on aggregated national data to inform aging and tribal health policies. Given the considerable racial, geographic, cultural, and socio-economic heterogeneity that characterizes India's tribal populations, aging trajectories and morbidity profiles are likely to vary across settings. National averages may therefore conceal localized vulnerabilities or overestimate disease burdens in specific regions. From a health equity perspective, these findings emphasize the need to prioritize small-scale, community-based primary research to generate region-specific evidence. Importantly, they call into question the appropriateness of a uniform “one nation, one policy” approach, advocating instead for context-sensitive, locally responsive policy frameworks that address region-specific morbidity burdens while remaining aligned with broader goals of healthy and equitable aging.

Aligning with existing literature (7, 11, 17), cardiometabolic morbidity found to be positively linked with functional, memory and sensory impairments. Similar to existing evidences (13, 17, 18), hypertension is found to be strongly associated with ADL limitation and memory impairment. The finding has been reinforced vascular dysfunction contributes to mobility limitations and early cognitive decline through pathways involving microvascular damage and reduced cerebral perfusion (13, 17, 18). In India, cardiovascular disease prevention and control are primarily addressed through the NPCDCS and the AB-HWCs, which have substantially expanded population-level screening for hypertension and diabetes, including in tribal and geographically remote areas. The routine integration of blood pressure screening, risk profiling, and essential drug provision at Health and Wellness Centres in India represents a major system strength for early cardiometabolic risk detection among tribal populations. However, the present findings suggest that policy focus must now move beyond detection alone. Given the strong linkage between hypertension and functional and cognitive impairment, especially in tribal older adults, there is a need to integrate routine functional, cognitive, and sensory screening into existing NCD platforms at the primary care level. Strengthening longitudinal follow-up, treatment adherence, and referral pathways- alongside culturally appropriate lifestyle counseling- could enhance the impact of existing programmes on healthy aging outcomes. Leveraging the established HWC platform to address cardiometabolic risk as a determinant of disability and cognitive decline may therefore be critical for reducing aging-related health inequities among tribal populations.

Consistent with previous literature (18, 19), overweight status is found positively linked with mobility limitation, memory and sensory impairment. These findings support growing literature emphasizing the multisystem consequences of excess adiposity, including chronic inflammation, oxidative stress, vascular compromise, and accelerated degenerative changes (18). Although WHR did not retain statistical significance in adjusted models, its association with visual impairment in unadjusted analyses suggests that abdominal adiposity may operate through complex metabolic or inflammatory pathways. This observation warrants more nuanced and context-specific investigation to elucidate the potential interplay between central obesity, cardiometabolic risk, and sensory decline in tribal aging populations.

Consistent with established gerontological literature (6, 8, 12), age is found to be significant predictor of multidomain impairments. The steep age gradients observed across functional, cognitive, and sensory domains reflect expected physiological decline but are likely intensified at pre-aging stage. Similar intensification of age-related decline has been documented in other Low- and Middle-Income Countries (LMICs), where substantial poverty rate and limited health infrastructure accelerate biological aging and reduce resilience in later life (20).

Socioeconomic factors were less consistently associated with impairment. Occupational status had limited influence overall. Although waged laborers had higher odds of memory impairment, this pattern may reflect cumulative exposure to strenuous or hazardous work, long-term fatigue, early frailty, or underlying social disadvantage (21). Educational attainment found to be strongly associated with impairment in unadjusted regression analyses, did not retain significance in adjusted models- likely due to uniformly in levels of schooling, which restricts meaningful variability for detecting gradients (11). Similar to existing literature (22), vegetarians were less likely to report mobility limitation compared with non-vegetarians. Given the unique cultural and economic context of dietary practices within this tribal population, this association may capture broader differences in lifestyle or socioeconomic profiles rather than nutritional effects alone. Further research is required to clarify the mechanisms underpinning this relationship.

Although the magnitude of multidomain impairment was lower among study population than that reported among tribal populations nationally, a substantial dual burden of early multidomain impairments and cardiometabolic morbidity among the study population could not be ignored (15). Despite this comparatively lower prevalence, substantial burden of functional, memory, and sensory impairments signals an urgent need for integrated community-based screening, strengthened outpatient care services, and preventive strategies addressing cardiometabolic risks from midlife onwards (23, 24). Moreover, the results emphasize the substantial heterogeneity within tribal populations and reinforce the importance of generating disaggregated, context-specific evidence to guide policies and interventions aimed at promoting healthy aging among marginalized communities.

### Policy implications

The findings of this study have important implications for public health policy and programme design aimed at promoting healthy aging among tribal geriatric populations.

1. The strong associations between hypertension, overweight, and multidomain impairments suggest that programmes such as NPCDCS should move beyond disease-specific cardiometabolic screening to incorporate routine assessment of functional ability, cognitive status, and sensory health among adults aged 45 years and older in tribal settings. Integrating multidomain aging screening within NCD platforms would facilitate early identification of disability risk and support preventive healthy aging trajectories.
2. The substantial burden of impairments highlights the need to strengthen AB-HWCs as comprehensive primary care hubs for healthy aging. HWCs should be equipped to provide integrated geriatric services, including functional and cognitive screening, risk counseling, referral pathways, assistive device linkage, and community-based rehabilitation tailored to tribal older adults.
3. Given the consistent role of overweight and central adiposity in predicting impairment, policies under the National Health Mission (NHM) should prioritize midlife interventions that promote healthy aging, including culturally appropriate nutrition counseling, physical activity promotion, and weight management strategies to prevent accelerated functional decline in later life.
4. Observed socioeconomic and occupational disparities reinforce the need to strengthen the Tribal Health Action Plan through aging-sensitive approaches, including improved outreach services, access to preventive and rehabilitative care, and social protection mechanisms that reduce disability-related vulnerability among marginalized tribal older adults.

### Limitations and strengths of the study

This study has certain limitations that should be acknowledged. The cross-sectional study design precludes causal inference between cardiometabolic risk factors and functional, cognitive, or sensory impairments; the observed associations may reflect reverse causality or shared underlying determinants. Additionally, functional, cognitive, and sensory impairments were assessed using self-reported measures, which may be subject to recall bias and misclassification, particularly for cognitive impairment that was not confirmed through objective clinical assessments. Additionally, the study was conducted in five tribal villages from a single district, which may limit generalizability to other tribal populations with different socio-cultural and environmental contexts.

Despite these limitations, the present study findings offer critical insights into the health challenges of a demographically marginalized tribal community. In particular, the present study found high prevalence of cognitive, hearing and visual impairment, functional limitation which usually hidden in large-scale survey findings. In addition, several modifiable risk factors of functional, sensory and cognitive impairments identified in this study, therefore the HWC could help identify at-risk individuals early. Addressing hypertension, overweight, and other modifiable risks must be prioritized to achieve healthy tribal aging population.

## Conclusion

The present study demonstrates a considerable burden of cognitive, functional, and sensory impairments among tribal adults aged 45 years and above in Gorakhpur, with nearly one-fifth reporting mobility, memory, visual, or hearing difficulties and more than one-quarter experiencing ADL limitation. Hypertension independently increased the likelihood of ADL limitation and cognitive impairment, while overweight status consistently predicted mobility, cognitive, visual, and hearing impairments. Advancing age showed the strongest gradient across all domains, with additional variation by occupation and dietary patterns. These findings have important public health policy implications for tribal geriatric populations. The clustering of multidomain impairments around modifiable cardiometabolic and anthropometric risk factors underscores the need to strengthen healthy aging strategies through early screening, preventive risk management, and integration of functional and cognitive assessment within existing primary care and tribal health programmes. Embedding comprehensive multidomain geriatric care into tribal health systems is essential to reduce disability burden and promote equitable healthy aging.

## Data Availability

The raw data supporting the conclusions of this article will be made available by the authors, without undue reservation.
